# The effect of video-based multimedia training on knowledge, attitude, and performance in breast self-examination

**DOI:** 10.1186/s12905-022-01877-w

**Published:** 2022-07-18

**Authors:** Zahra Karimian, Roya Zare, Nahid Zarifsanaiey, Nasim Salehi

**Affiliations:** 1 Department of E-Learning in Medical Sciences, Virtual School and Center of Excellence in E-Learning, Shiraz University of Medical Sciences, Shiraz, Iran; 2 Virtual School and Center of Excellence in E-Learning, Shiraz University of Medical Sciences, Shiraz, Iran; 3 Faculty of Health, Southern Cross University, Gold Coast, QLD Australia

**Keywords:** Breast neoplasms, Breast self-examination, Training, Chronic disease, Knowledge, Attitude, Performance, Health promotion, Women, Iran

## Abstract

**Background/Objectives:**

Breast neoplasm is one of the most common cancers in Iranian women due to the late diagnosis. Awareness of breast neoplasm and using Breast Self-Examination (BSE) assist in the early detection and treatment of cancer. This study examined the effectiveness of video-based multimedia training versus face-to-face training in awareness of breast neoplasm and BSE and possible factors affecting their effectiveness.

**Methods:**

This research was a pre-test, a post-test experimental study comparing the knowledge, attitude, and performance of women about BSE across two training intervention groups (face-to-face versus video-based multimedia). The study was conducted at Shiraz University of Medical Sciences (SUMS), and 100 women between 20 to 60 years old were allocated to each intervention group via multi-stage cluster sampling (n:110). Three valid and reliable researcher-made questioners were used. Data were analyzed using SPSS 24 with independent *t*-test, paired *t*-test, and ANOVA.

**Results:**

Both video-based multimedia and face-to-face training methods significantly increased the participant's knowledge, attitude, and skills about breast self-examination (*P* < 0.001). In the sub-categories, the results showed that the face-to-face training improved negligence and forgetfulness in applying BSE (*P* = 0.03) and correcting or modifying the previous knowledge around the issue (*P* = 0.02). The effect of the video-based method on participants with university education was more than on non-university (*P* = 0.04).

**Conclusion:**

Incorporating video-based multimedia training in awareness of breast neoplasm and breast self-examination provides an easy, flexible, and affordable way for detection, particularly considering crisis restrictions. This can be of particular attention in more populated, developing/low-income countries and rural and remote areas to enhance equitable access to training and facilitation diagnosis and treatment if applicable.

## Introduction

There is an increasing trend in chronic diseases, such as cancer, with an estimated 6.7 billion [1]. This can result in high costs and a burden on the healthcare system, consumers, family members, and society, particularly in comorbidities. There is a shift towards prevention through health promotion approaches such as healthy lifestyle behaviours and early diagnosis to decrease the cost and burden of chronic diseases. However, implementing effective and sustainable diagnoses and preventive strategies to delay chronic diseases can be challenging and require strategic planning [1].

Breast neoplasms are one of the most common cancers in women [2] and ranked fifth as the leading cause of death in cancer-related diseases, with 1.2 million new cases per year [3–5]. It is the leading cause of death in women aged 41–44 [6] and includes 43% of the whole percentage of cancers [7, 8]. Based on GLOBOCAN, the rate of breast neoplasm will increase from 24% in 2018 to 46% in 2040 [9]. According to WHO [10], breast neoplasm has an increasing daily trend (11.6%) and death rate (6.6%) and is located as second cancer in regards to the death rate, particularly in women more than 50 years old [10].

Cancer is the third leading cause of death in Iran [11], including breast neoplasm (approximately 33 in 100,000 women). The rate of breast neoplasm is higher in Iran than the global rate (ASR: 31), and it is estimated to be doubled by the end of 2030 (ASR = 70) [12]. The rate of breast neoplasm is increasing in women under 50 years old, in Iran, in comparison with other developed countries, indicating the onset at an earlier age [13, 14]. The average beginning has been reported at age 34.5, which is lowers than the global average [15]. Although, based on the statics, the number of breast neoplasms seems higher in developed countries, the death percentage is higher in developing countries [16], indicating the importance of early diagnosis and prevention strategies [17]. Hence, enhancing awareness for early diagnosis of breast neoplasm is paramount [18], as there can be a 90% survival rate [19] due to the advancement in diagnosis and treatment [20].

There can be diverse methods for the diagnosis of breast neoplasms world wild, including mammogram, ultrasound, biopsy, and MRI. Breast Self-Examination (BSE) can be considered a complementary method in assisting women in reporting any unusual changes early on [11, 21]. Overall, breast screening expenses are said to be very high in Iran. In 2013 847,544.96 US dollars were spent on screening programs for women aged 25–34 and above 35 in Iran [11]. Although BSE can not replace other precise screening methods such as mammography and ultrasound, it can be considered a complementary approach to early diagnosis and decrease the financial burden of breast neoplasms screening (*24). Undoubtedly, clinical screening by healthcare professionals can be more reliable. BSE is still considered very effective due to its relatively satisfactory diagnosis rate (65%) [22]. This can be of particular attention in developing countries due to its affordability, flexibility, and ease of use [23]. The unfortunate relatively high percentage of the population, particularly in developing countries, may not know how to practice BSE [24–29].

Currently, there are relatively low numbers of women applying for BSE in developing counties such as Iran, which can be due to different factors, such as lack of literacy, lack of awareness/training, fear, and cultural and social taboos [19, 24, 28]. The other relevant variables impacting BSE can be marital status, age, socioeconomic status, education, and religious affiliation [2, 30–32]. For example, the frequency of BSE can increase with age [32]. Implementing BSE training and awareness can have a primary role in understanding the BSE process [2, 33]. There is a significant association between Knowledge of BSE practices. Knowledge has been highlighted as a critical indicator of women's behaviour and action around BSE [2, 34, 35].

According to a review paper on BSE training, there can be diverse ranges of facilitators and barriers impacting practical training on BSE. For example, participants' motivation for learning, socio-demographic factors, and the societies' social, cultural and financial situation affect the success of BSE training. Other impacting factors can be flexibility in the training (not being restricted to a specific time and place), effective communication and relationship with healthcare providers and resources, and providing the proper training for the right population. This study emphasized access to different training approaches, including multimedia training, to enhance flexibility and effectiveness and make the training more individualized. It was also suggested to understand the barriers to using multimedia approaches, such as age (older age range), low level of education and literacy, lack of support and resources, and lack of follow-up [36].

There have been diverse research on the benefits of BSE training programs, regardless of their type [33, 36–38]. However, most studies regarding BSE awareness have focused on face-to-face training. The majority have been descriptive, without any specific interventions, or including only one intervention group, without comparison.

Limited studies considered the social media approaches toward training and awareness of BSE [33]. In addition, limited studies have compared the effectiveness and efficiency of different training approaches, such as face-to-face, blended, and multimedia training in BSE. Multimedia approaches can be paramount in the learning process, considering the increasing trend toward online education and technology to make the training more personalized and effective. It is beneficial for policy and practice changes to examine the effectiveness and efficiency of BSE approaches [33]. This can be of particular attention in countries with limited resources and remote and rural areas [2], highlighting the "no one size fits all" approach.

Since face-to-face training can be time-consuming, restrictive, and costly, it will be more feasible and efficient to turn into other alternatives, such as multimedia training programs [39]. Video-based multimedia training platforms can be of particular attention due to their variation, affordability, high level of engagement, and involving different senses [39]. In addition, multimedia training can be more flexible due to asynchronous and synchronous approaches and can be adjusted based on the audiences' capabilities to be more personalized [40]. This is of particular attention during crises to leverage technologies to improve the training infrastructure, normalize virtualization, and enhance flexibility [41]. Overall, two critical literature gaps resulted in the current study. First, although there has been plenty of research on face-to-face and multimedia training separately, there have been limited studies comparing these two. This is particularly important in developing countries because of the limited financial resources. Second, as highlighted, there is a high economic cost due to the unnecessary screening referrals in Iran, which can be prevented by enhancing alternatives such as BSE. In the current study, we aimed to compare video-based multimedia training using social media (WhatsApp) with face-to-face training to examine the enhancement in breast neoplasm awareness(Knowledge), Attitude and BSE performance across the two programs.

## Method

### Research design

The present pretest–posttest experimental study was conducted on two training programs (video-based multimedia versus face-to-face) regarding breast neoplasm awareness and BSE. The study population consisted of all women attending their general practitioner in 2019 in Shiraz, Iran.

### Sampling

Based on the consultation sessions with research experts and similar works in 2012 [42], 44 participants were required in each group. Considering a confidence interval of 95% and a 20% attrition rate, a total of 55 participants were evaluated in each group.

$$n = \frac{{\left( {z_{{1 - \frac{\alpha }{2}}} + z_{1 - \beta } } \right)^{2} \left( {\delta_{1}^{2} + \delta_{2}^{2} } \right)}}{{\left( {\mu_{1} - \mu_{2} } \right)^{2} }}$$

$$\mu_{1} - \mu_{2} = 0.96 \quad \alpha = .05 \quad 1 - \beta = 0.9$$

$$z_{{1 - \frac{\alpha }{2}}} = 1.96 \quad z_{1 - \beta } = 1.3 \quad S_{1} = 1.79 \quad S_{2} = 0.82 \quad \mu_{2} :9.06 \quad \mu_{1} :8.1$$

Random multiple cluster sampling was used. First, one of the two key health centres in Shiraz was randomly selected, then all the healthcare settings under that health centre were labelled a number, and then ten were chosen randomly. A total of 10–11 participants were randomly selected from each healthcare setting (N = 110). Participants were categorized based on their socio-demographic status to include a diverse range of participants. After random selection, participants were contacted for some primary screening (e.g., their interest in being involved in the study, previous participation in any relevant training, and access to computers and laptops for video-based multimedia training).

**Fig. 1:**
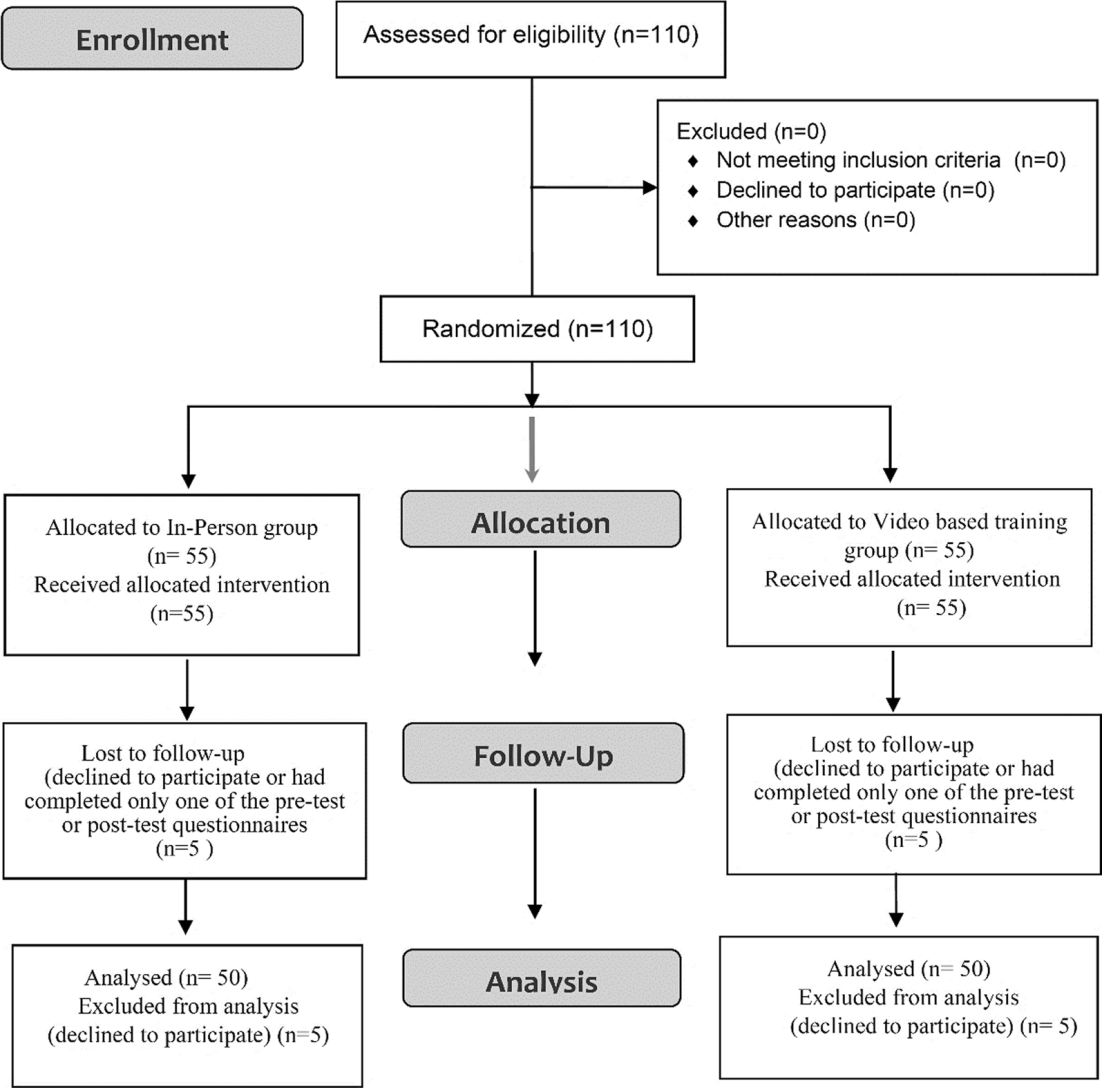
The participants” recruitment flow diagram

Women were included if they registered under healthcare centres in Shiraz, aged 20–60, did not have psychological illnesses, and had no previous participation in any relevant training programs on BSE. Then 110 participants were randomly divided into two intervention groups (Fig. 1). Participants were excluded if they decided not to continue the project and did not complete the survey.

### BSE training content development and delivery

The educational content for both interventions included two key sections. (1) Theoretical training, comprising four parts, including Breast neoplasm, Prevention of Breast neoplasm, Concepts and Methods of BSE. (2) Practical section, which showed the application and demonstration of BSE steps (Table 1). According to World Health Organisation, the content was developed according to reputable sources, such as previous literature around the topic and existing protocols in the Iranian health ministry on BSE. In addition, we had multiple consultations with experts and specialists in breast neoplasm.

**Table 1:** Key training categories for BSE content development

Sections	Titles	Sub-titles
Theoretical	Introduction to breast neoplasm	Prevalence and incidence of breast neoplasm
		Risk factors of breast neoplasm
	Prevention of breast neoplasm	Self-care factors of breast neoplasm
		Diagnosis of the breast neoplasm
	Concepts and methods of breast self-examination	Introduction to BSE
Practical	Demonstration of BSE steps	Practical steps of BSE

We used an active and cooperative learning approach in the face-to-face training group. Educational content was delivered via four short lectures (Each lecture is about 30 min) and presentations, including PowerPoints, as well as questions and answers at the end of the session to enhance the engagement of the participants with the facilitator and peers. After each session, participants asked questions about the content to assess their understanding of the content. Practical learning was applied in two ways, including active practising on medical moulage and BSE mannequin (in Skill Lab) and participants (in private, based on their preference). The whole session was about 5–6 h, including 2.5–3 h of training and 2.5–3 h of practising and answering sessions to engage in the content and practice BSE.

The content in the multimedia training group was similar to the face-to-face group. For delivery purposes, first, the content was structured in four PowerPoint files, based on the critical categories mentioned in Table 1. Then, using iSpring Suite software version 8, the voice was added, and after that, using Quiz software, other layers, such as animation and content segmentation, were added (Table 2).

**Table 2:** Comparison of the content and practice via face-to-face and online groups

Criteria	Content	Face to face	Online
Scientific content	Common cancers in women, breast neoplasm, early diagnosis of breast neoplasm, confronting and managing breast neoplasm	✓	✓
	Assessing and confirming the validity and reliability of the content via	✓	✓
Multimedia elements	Text	✓	✓
	Images	✓	✓
	Voices	✓	✓
	Consider the segmentation and animation of text, sound, and images	✓	✓
	Consider the multimedia criteria for the content	✓	✓
Practice and exercise	Presenting practical steps related to the content (BSE)	✓	✓
	Practising the BSE under the supervision of the mentor	✓	–
Formative Assessment	Provision of questions after finishing each section	✓	✓
Discussion (Q&A)	Asking questions by participants during training	✓	–
	Asking questions participants after finishing the lessons	✓	✓

In building the multimedia, a micro-learning approach was used. Overall, there were four training videos, each lasting ≤ 10 min. The four videos in MP4 format include an introduction to breast neoplasms (10 min), prevention of breast neoplasms (7 min), concepts and methods of breast self-examination (7 min), and demonstration of BSE (6 min). The size of videos was expressed by HandBrake software and shared with participants on WhatsApp. All participants were added to the WhatsApp group in advance. There was a facilitator to respond and give feedback to the questions if needed. At the end of each section, participants answered some Multiple Choice Questions(MCQ) around the topics. Multimedia videos were developed and assessed based on *Richard Mayer's* Multimedia Principles [43–45] and by experts in the area of e-content development in the *Center of Excellence in E-Learning(CEEL)* at Shiraz University of Medical Sciences (SUMS). Relevant checklists were used for developing and quality assurance of the multimedia videos. All the training delivery and assessments were done by experts in the area who were not a part of the research. This strengthens the rigour and incredibility of the process.

### Intervention procedure

#### Pre-test

Pre-test was conducted in July 2019 by randomly allocating participants to each group. Before starting the training, demographic information was achieved. In addition, the questions related to knowledge and attitude were completed. Using the relevant checklist and observation, participants assessed BSE application by participants.

Training intervention: Intervention in both groups was done for two months, in 2019, across different groups. There were four workshops for 55 participants in each intervention group (of 4 groups, with 10–15 participants for each intervention). Each group had a one-day training comprising theoretical and practical BSE training. Face-to-face training was done as a daily workshop, including 5–6 h. The academic section was done via presentation and lectures, including pictures, moulage, BSE mannequin, and Q&A sessions (three hours in duration). The practical section includes applying the BSE privately with the facilitator to confirm if the process has been done correctly. The training team included a specialist in midwifery and breast neoplasms, as well as six facilitators and tutors in health promotion, midwifery, and nursing. They all had at least ten years of work experience in BSE education. First, a WhatsApp group was created in the multimedia group, and participants were added. Then, four videos were shared in the group every three days. After sharing each video, some questions assessed participants' understanding of the content. There were Q&A sessions, providing opportunities for participants to discuss their questions and engage with their peers and facilitators. The duration was 15 days.

#### Post-test

**Fig. 2:**
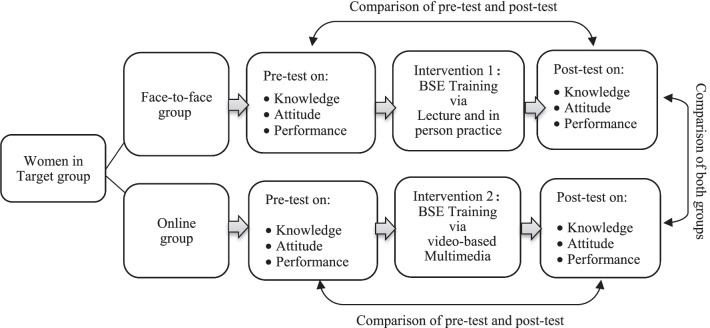
Intervention procedure for both control and intervention groups

After finishing the training, participants were asked to do the post-test on knowledge and attitude (in their group). They also applied the BSE performance in healthcare settings under the supervision of facilitators (who were blinded to the study). In addition, three months after the training, all participants were contacted to complete the post-intervention questionnaires and the BSE. A total of 100 women participated in the post-intervention survey and BSE. In each group, there were an overall number of 5 attritions that either did not attend the session or did not complete the surveys fully. These participants were excluded from the project, resulting in an overall of 100 participants. The process of educational intervention in both groups is shown in Fig. 2.

#### Research instruments

Three essential tools were used, and their content validity was assessed by ten experts in the area, including Obstetrics and Gynaecology (n = 5), midwifery (n = 3), and education (n = 2). The instruments include three sections:

##### Knowledge

The researcher-made questionnaire, including 20 MCQ questions, comprises five categories: Incidence and prevalence of the disease (5); risk factors (4); self-care factors (3); diagnosis (4), and BSE steps (4).

Multiple-choice questions were created based on the main references, and existing protocols in the *Ministry of Health, Treatment and Medical Education* of Iran, which follows WHO protocols. In addition, a specialist in women's health with expertise in breast neoplasm was responsible for developing the questions. The questions were also sent to another ten specialists in the same area to assess the content validity of the questions. Experts' views completed face validity. Revisions were applied to enhance the clarification and simplicity. Content validity was done via engaging specialists in Obstetrics and Gynaecology(4), PhD in midwifery(1), MSc in midwifery(2), Family Health(1) and PhD in Education(2). Content Validity Index (CVI) [relevance (94%), simplicity (93%), and clarification (%92.5)]. Content Validity Ratio (CVR) was used to assess the necessity of the questions (85%). The cut-off point for knowledge assessment was a minimum of 60%, indicating a score of 12 out of 20.

##### Attitudes

The questionnaires created by Zare et al., Which were reported in an article two years later in 2021 [19], were used for assessing the attitudes towards breast neoplasm prevention and BSE. This includes 12 questions in 5 categories around norms and beliefs (3), fear of breast neoplasm (3), Uncomfortable with doing BSE (2); Negligence and/or forgetfulness (2); Previous Knowledge. (3). Likert scale was used (1 disagree to 5, completely agree). A total of 10 experts in a study by Zare et al. assessed the content validity of the questionnaire via CVI related to relevancy (0.99%), simplicity (0.98%), and clarification (0.99%). The necessity of items was assessed via CVR (%78). Reliability was evaluated via internal consistency of the questions, using alpha Cronbach (84%).

##### Performance

A research-made checklist based on WHO guidelines, including 15 questions, was used, covering five categories such as Performing for the BSE (2); hands and fingers position (2); patterns of BSE (3); BSE Various positions (2); BSE practical steps (6). The scores included entirely right (3), relatively right (2), and wrong (0). The checklist suggested by Abera (2017) was used in framing the performance section. Content validity was done via engaging specialists in Obstetrics and Gynaecology (4), PhD in midwifery (1), MSc in midwifery (2), Family Health (1) and PhD in Education (2). Based on the outcome of the training, including content table, Content Validity Index (CVI) [relevance (%94.67), simplicity (96%), and clarification (%94)]. The cut-off point for performance assessment was a minimum of 60%, indicating a score of 18 out of 30, considering the importance of performance. Those with a low score on each item were encouraged to repeat the performance to achieve the required score, ensuring that they had applied the BSE correctly. When assessing the participants' performance, DOPS (Direct Observation of Practical Skills) was used to provide further feedback for corrections.

Socio-demographic questions asked from participants included age, marital status, education, receiving any other formal and informal training, illness history in the individual, and family.

#### Data analysis

Paired *t*-test was used for comparing pre and post-test surveys. An independent sample *t*-test was used to compare the means of two interventional groups. ANOVA and an independent-sample *t*-test were used to compare the differences across the socio-demographic groups.

#### Ethical consideration

This research was approved by SUMS (IR.SUMS.REC.1398.101). All the participants completed the consent form based on the SUMS ethical committee. The results were analyzed anonymously, and a report was provided to the health-related authorities for some practical actions.

After finishing the research, participants who attended the face-to-face training were provided with videos on multimedia training. Those who participated in the multimedia training were also allowed to repeat the face-to-face training if they wished.

## Results

An overall 100 participants (out of 110) answered the questions and participated in the training interventions (50 in each group). Descriptive findings are summarised below (Table 3).

**Table 3:** Socio-demographic characteristics in the face to face and online groups

Variables	Sub-category	Face to face	Online
Age	Highest age	60	55
	Least age	22	20
	Average	39.2 ± 7.4	37.9 ± 7.10
	20–30	6 (12%)	7 (14%)
	31–40	21 (42%)	26 (52%)
	41–50	21 (42%)	14 (28%)
	51 ≥	2 (4%)	3 (6%)
	Total	50 (100%)	50 (100%)
Marital status	Single	5 (10%)	6 (12%)
	Married	45 (90%)	44 (88%)
	Total	50 (100%)	50 (100%)
Education	Primary and secondary school	2 (4%)	0(0%)
	Diploma	30 (60%)	29 (58%)
	Bachelor	18 (36%)	19 (38%)
	Master and higher	2 (3.9%)	2 (4%)
	Total	50 (100%)	50 (100%)
Employment status	Employed	42(84%)	40(80%)
	Unemployed	3(6%)	2(4%)
	Missing	5(10%)	8(16%)
	Total	50 (100%)	50 (100%)
Breast neoplasm history in the family	Yes	2 (4%)	3 (6%)
	No	48 (96%)	47 (94%)
	Total	50 (100%)	50 (100%)

### Knowledge

Results showed video-based multimedia had significant improvement in the knowledge of participants. Knowledge score was low in the pre-test survey in both groups. However, this has increased in post-test in both groups (*P* < 0.001), indicating the effectiveness of both training interventions. The pre-test scores regarding knowledge in both face-to-face and video-based multimedia interventions showed no significant difference (*P* = 0.91). Although the overall post-test average was higher in face-to-face training than in video-based multimedia training (*P* < 0.001), this was only significant in regards to one of the sub-categories (incidence and prevalence of the breast neoplasm) (*P* < 0.001) (Table 4).

**Table 4:** Comparison of Knowledge dimension scores across the face-to-face and online groups

Components	Groups	df	T	Average		*P*-value
				Pre-test	Post-test	
Comparison of the total score (0–20)	Face to face	50	18.97	8.74 ± 2.75	16.92 ± 2.53	< 0.001
	Online	49	14.77	8.80 ± 2.35	14.70 ± 2.69	< 0.001
	Between-group comparison	99	–	*P* = 0.91	*P* < 0.001	–
*Comparison of sub-categories of each training section*						
Prevalence and incidence of the BC (0–5)	Face to face	50	15.20	1.39 ± 1.20	4.27 ± 0.89	< 0.001
	Online	49	11.37	1.44 ± 0.76	3.22 ± 1.05	< 0.001
	Between-group comparison	99	–	*P* = 0.81	*P* < 0.001	–
Risk factors (0–4)	Face to face	50	7.75	2.07 ± 3.29	3.29 ± 0.75	< 0.001
	Online	49	7.21	2.18 ± 0.94	3.20 ± 0.83	< 0.001
	Between-group comparison	99	–	*P* = 0.61	*P* = 0.55	–
Self-care factors (0–3)	Face to face	50	7.65	1.13 ± 0.77	2.26 ± 0.86	< 0.001
	Online	49	8.06	1.14 ± 0.80	2.26 ± 0.69	< 0.001
	Between-group comparison	99	–	*P* = 0.98	*P* = 0.87	–
Diagnosis of the breast neoplasm (0–4)	Face to face	50	14.30	1.41 ± 0.89	3.47 ± 0.73	< 0.001
	Online	49	12.76	1.40 ± 0.92	2. 38 ± 0.80	< 0.001
	Between-group comparison	99	–	*P* = 0.94	*P* = 0.55	–
BSE steps (0–4)	Face to face	50	5.58	2.72 ± 1.11	3.54 ± 0.75	< 0.001
	Online	49	3.55	2.64 ± 0.96	3.21 ± 0.86	< 0.001
	Between-group comparison	99	–	*P* = 0.68	*P* = 0.48	–

### Attitudes

Results showed video-based multimedia training had significant improvement in the attitude of participants. There were no significant differences in the pre-test scores of the two intervention groups (*P* = 0.49), and the attitudes score was low in the pre-test survey in both groups. However, this has increased in post-test in both groups (*P* < 0.001), indicating the effectiveness of both training interventions. Also, there were no significant differences across face-to-face and video-based training methods (*P* = 0.08), but in the sub-categories, the results showed that the face-to-face training showed more effectiveness in improving negligence and/or forgetfulness in applying BSE (*P* = 0.03); as well as in correcting or modifying the previous knowledge around the issue (*P* = 0.02). (Table 5).

**Table 5:** Comparison of Attitude dimension scores across the face-to-face and online groups

Components	Groups	df	T	Average		*P*-value
				Pre-test	Post-test	
Comparison of the total score of attitude (1–5)	Face to face	46	6.05	3.15 ± 0.35	3.56 ± .62	< 0.001
	Online	40	4.05	3.07 ± 0.37	3.72 ± 0.68	< 0.001
	Between-group comparison	92	–	*P* = 0.49	*P* = 0.08	–
Comparison of sub-categories of each training section						
Norms and beliefs	Face to face	49	8.65	3.52 ± 0.34	4.16 ± 0.59	< 0.001
	Online	44	16.45	3.40 ± 0.45	4.22 ± 0.52	< 0.001
	Between-group comparison	94	–	*P* = 0.14	*P* = 0.76	–
Fear of breast neoplasm	Face to face	48	2.43	2.95 ± 0.28	3.15 ± 0.78	0.02
	Online	43	0.43	2.93 ± 0.50	3.04 ± 0.75	0.24
	Between-group comparison	94	–	*P* = 0.89	*P* = 0.42	–
Uncomfortable to do BSE	Face to face	49	6.41	3.16 ± 0.47	3.81 ± 0.77	< 0.001
	Online	45	8.18	3.13 ± 0.51	3.89 ± 0.89	< 0.001
	Between-group comparison	98	–	*P* = 0.74	*P* = 0.60	–
Negligence and/or forgetfulness	Face to face	47	0.86	2.97 ± 0.48	2.99 ± 1.08	0.93
	Online	44	2.5	2.57 ± 1.41	2.90 ± 0.50	0.01
	Between-group comparison	95	–	*P* = 0.82	*P* = 0.03	–
Previous knowledge	Face to face	48	4.37	3.17 ± 0.64	3.71 ± 1.77	< 0.001
	Online	44	1.79	2.94 ± 0.73	3.18 ± 1.27	< 0.001
	Between-group comparison	94		*P* = 0.17	*P* = 0.02	

### Performance

Results showed video-based multimedia had significant improvement in the performance of participants. The score was low in the pre-test survey in both groups. However, this has increased in post-test in both groups (*P* < 0.001), indicating the effectiveness of both training interventions. There were no significant differences across the two intervention groups regarding performance for the pre-test **(***P* = 0.26). There were no significant differences concerning the effectiveness of the two interventions (*P* = 0.38). In none of the sub-categories, there was no significant difference between the two educational methods (*P* > 0.05) (Table 6).

**Table 6:** Comparison of Performance dimension scores across the face-to-face and online groups

Components	Groups	df	T	Average		*P*-value
				Pre-test	Post-test	
Comparison of the total score (0–30)	Face to face	50	13.51	4.11 ± 2.25	22.71 ± 7.34	< 0.001
	Online	49	19.35	5.08 ± 3.60	21.48 ± 5.08	< 0.001
	Between-group comparison	99	–	*P* = 0.26	*P* = 0.38	–
*Comparison of sub-categories of each training section*						
Performing the BSE (0–4)	Face to face	50	13.60	0.82 ± 0.55	2.90 ± 1.20	< 0.001
	Online	49	13.71	0.84 ± 0.52	2.88 ± 1.31	< 0.001
	Between-group comparison	99	–	*P* = 0.97	*P* = 0.93	–
Hand and fingers position while doing BSE (0–4)	Face to face	50	12.50	1.47 ± 0.94	3.31 ± 0.90	< 0.001
	Online	49	17.73	1.38 ± 0.0	3.34 ± 1.51	< 0.001
	Between-group comparison	99	–	*P* = 0.61	*P* = 0.89	–
Patterns of the BSE (0–6)	Face to face	50	10.53	0.92 ± 1.41	4.35 ± 1.70	< 0.001
	Online	49	14.48	1.28 ± 1.26	4.33 ± 1.77	< 0.001
	Between-group comparison	99	–	*P* = 0.18	*P* = 0.92	–
Various positions (0–4)	Face to face	50	11.69	0.43 ± 0.87	3.13 ± 1.21	< 0.001
	Online	49	14.72	1.04 ± 0.92	3.20 ± 1.16	< 0.001
	Between-group comparison	99	–	*P* = 0.01	*P* = 0.79	–
BSE practical steps (0–12)	Face to face	50	13.50	0.47 ± 2.02	9.03 ± 3.75	< 0.001
	Online	49	16.36	0. 56 ± 1.45	7.74 ± 2.20	< 0.001
	Between-group comparison	99	–	*P* = 0.80	*P* = 0.65	–

However, it was applicable to make a comparison in regards to education and age variables. 

### Education

The education level of participants was divided into university versus non-university education. There was a significant difference in educational status and video-based multimedia training in *Knowledge* (*P* = 0.04). Those with university degrees had a higher level of knowledge in regards to breast neoplasm awareness and BSE in the video-based multimedia group. There were not any significant differences in regards to education in face-to-face training. There was a significant difference in educational status, face-to-face training, and *attitude* (*P* = 0.03). It is important also to mention that the pre-test score of the face-to-face group regarding attitude was also higher than the video-based multimedia group (*P* = 0.01), which might have impacted the post-test score (Table 7). There were no significant differences across the two groups regarding the performance dimensions and education.

**Table 7:** Differences across knowledge and attitude dimensions of BSE training, based on education

Components	Groups	N	Average		*P*-value
			Pre-test	Post-test	
Knowledge * Education (Score: 0–20)					
Face-to-face	None-academic	32	8.59 ± 2.74	16.43 ± 2.65	< 0.001
	Academic	18	9.11 ± 2.86	17.72 ± 2.16	< 0.001
	Between-group comparison	–	*P* = 0.53	*P* = 0.08	–
Online	None-Academic	29	8.48 ± 2.26	14.03 ± 2.87	< 0.001
	Academic	21	9.23 ± 2.46	15.61 ± 2.18	< 0.001
	Between-group comparison	–	*P* = 0.26	*P* = 0.04	–
Attitude * Education (Score: 1–5)					
Face-to-face	None-academic	29	3.05 ± 0.35	3.39 ± 0.60	< 0.001
	Academic	18	3.24 ± 0.29	3.78 ± 054	< 0.001
	Between-group comparison	–	*P*** = **0.01	*P*** = **0.03	–
Online	None-academic	26	3.02 ± 0.32	3.26 ± 0.57	< 0.001
	Academic	18	3.20 ± 047	3.44 ± 083	< 0.001
	Between-group comparison	–	*P* = 0.14	*P* = 0.39	–

Regarding the education variable, either the relationship with training was not significant, or due to limited responses in each group, we could not do compassion or apply the multivariate analysis.

### Age

Regarding age, the categories were 20–30, 31–40, and more than 40 years old. Findings showed no significant differences across the two interventions based on these age categories.

In the current study, we could not examine the relationship between marital status and breast neoplasm history in a family with BSE. Table 3 shows only 5–6 participants were reported as single, and 44–45 were married. Only 2–3 participants were informed with breast neoplasms family history, compared to 47–48 participants without a family history. Regarding the employment status, only 2–3 participants were reported with employment status, compared with 40–42 participants without employment. In addition, some of the participants did not reply to these demographic variables.

## Discussion and conclusion

The finding showed significant results in using video-based multimedia training in awareness of breast neoplasm and SBE. In addition, this study highlighted the importance of mobile and using social media (WhatsApp), which enhanced the training's flexibility and ease of use. This allowed excellent retention and engagement of participants across the video-based multimedia training. Overall, the finding highlighted that both face-to-face and video-based multimedia approaches are beneficial in BSE. The effectiveness was significant for both interventions across the three dimensions: knowledge, attitudes, and performance.

Regarding the knowledge dimension, pre and post-test results showed the effectiveness of both face-to-face and video-based interventions in improving the knowledge of individuals about breast neoplasm and BSE. Other research showed similar results [25, 28, 37, 46]. Overall, video-based and face-to-face training (blended learning) provides an ideal and complementary atmosphere for enhancing knowledge and dramatically impacts the practical learning journey [47]. When comparing multimedia learning with face-to-face, some studies showed that face to face method had been considered superior, which can be due to the high level of engagement between students, their facilitators, and peers [38, 48, 49]. Another reason can be the practical work done on mannequins, which can result in direct correction and a more straightforward learning process [38, 49]. The mannequin can have multiple other benefits, including developing professional identity, enhancing caring and communicative skills, the experience of a sense of realism and behaving as nurses, and improving the social learning environment [50].

Regarding the attitude dimension, pre and post-test results showed that both interventions' attitudes increased post-test compared to the pre-test. Overall, there was a low score in the attitude pre-test, even for those women with high knowledge/awareness of breast neoplasm prevention and early diagnosis [5, 37, 51]. Training via mobile phones had a more significant role in improving the attitudes of individuals [33], which can be due to the flexibility and more engaging nature of mobile phones rather than other multimedia platforms such as disks, which are more passive and less interactive. In addition, the short messages via mobile phone had more impact than comparison with a pamphlet [52], which can be again due to its flexibility, quick access, and instant feedback. Engaging communication with facilitators and pamphlets is superior to only pamphlets [53]. In the current study, it has been shown that face-to-face training was more applicable in improving two items—negligence and/or forgetfulness to apply BSE.

Regarding performance dimension, pre and post-test results showed a low score in performance pre-test, even for those women with high knowledge/awareness of breast neoplasm prevention and early diagnosis [8, 27, 28, 30, 31, 37, 54]. Face-to-face and video-based multimedia training improved women's performance in BSE [18, 42, 55] with no significant differences [52, 56]. Some studies showed a more substantial result in face-to-face learning [57] or blended learning than only online learning [53].

Overall, there were no significant results regarding age and training in the current study, which could be due to the relatively low numbers of participants. There have been mixed findings across the literature, indicating no difference [58], better performance in the older population concerning BSE [32, 57], or better performance in younger women, less than 39 [2]. These differences can be related to the differences across other populations and other confounding variables such as marital status and education. The justification for more engagement of young women is possibly the higher level of engagement on social events and social media [2]. In addition, our study showed that using multimodal video-based training, particularly those with a higher level of education, particularly those with university qualifications, had a much better performance in regards to BSE. This is aligned with other studies [30, 47]. This can be due to their experiences and knowledge of using technologies. Not only the level of education but the type of education also can impact the usage of multimedia training. For example, those with a background in health-related disciplines may have a higher self-consciousness towards their health and wellbeing [26].

Although face-to-face training can provide more opportunities for instant feedback and sharing of information, online education can be more flexible and cover a larger population. Online training can provide more options, particularly during crises such as Covid-19, to provide a more accessible, equitable, and affordable health knowledge outcome in both metropolitan and regional areas. This is of particular attention in developing countries considering the high population. Although one of the reasons for the lack of adherence to BSE is negligence and/or forgetfulness, video-based multimedia training via mobile phone can be beneficial to remind women of regular BSE.

This study provides implications and/or recommendations for policymakers, practitioners, researchers, and educators. First, a lack of health literacy and evidence-based information on breast neoplasm, as well as wrong attitudes around it, can play a significant role in delaying the BSE and failure to receive the proper diagnosis at the right time for on-time prevention, early diagnosis, and treatment [2]. Improving the knowledge level can significantly improve women's attitude toward Breast neoplasm and BSE and subsequently affect their performance. This can enhance women's knowledge of the issue on a larger scale and evaluate the consequent changes, such as help-seeking behaviours. It is crucial to explore the practical barriers impacting the knowledge, attitudes, and practice across different levels in an ecological framework. At the individual level, this can be age range, socioeconomic status (e.g., education), lack of knowledge and awareness around the techniques for BSE, assuming that there is no need for screening/not having symptoms, lack of privacy at home, not feeling comfortable, and also some socio-cultural factors at the societal levels [2, 24, 28, 36]. However, the other factors at the macro level are paramount as well. Educational level impacts video-based multimedia training, indicating that women with higher educational levels can be targeted for this. However, other factors also need to be considered to see how the level of engagement can increase, such as acceptance of online education, motivation, and social and cultural factors. This can include the healthcare systems, policies, and practices and how they support women in terms of informational and instrumental support across different areas, including regional and rural areas. The health promotion implication is to develop a social and culturally appropriate educational platform for diverse ranges of population across different age groups and geographical regions [2, 36].

Second, the scattered distribution of the population and the inconvenience of traveling for face-to-face training and diagnosis can make it challenging to provide equitable and uniform training and diagnosis. On the other hand, poor computer literacy can make it difficult to use online training and access information. There is a need to develop flexible training platforms and/or tools to meet the practical needs of women (across different geographical locations) in terms of enhancing health literacy and their BSE [2, 36]. It is recommended that educators consider women's experiences of video-based multimedia training, its impact, and barriers and use a personalized approach to training based on socio-demographic factors. Including more advanced techniques, such as simulation programs, can make learning more authentic, active, and engaging. Furthermore, the socio-cultural background of the societies and how they can impact the training need to be considered, as their possible impact on knowledge, attitude, and performance.

Third, by enhancing the literacy level around the issue and its impact on improving knowledge, attitude, and performance, women will be more conscious of the cancer warning signs, diagnosis, and the treatment process [30, 36]. Great attention is required to enhancing the tools for improving women's health literacy, particularly in regional areas, regarding the warning cancer signs. Furthermore, closer monitoring processes can be helpful to see how the training via different platforms/devices improves women's awareness of the issue and which tool can be more effective and, at the same time, efficient and enhance equitable access across different populations.

Fourth, it is recommended to conduct further studies comparing different online training approaches to breast neoplasm awareness and BSE regarding their effectiveness and efficiency. In addition, it is suggested to achieve a more in-depth understanding of women around the online educational platforms and their preferences. Furthermore, studies are required around how online training can be adjusted based on the geographical situation (metropolitan versus remote and regional areas), and how there can be more flexibility to access the online content for more equitable access across different populations. Finally, further studies with a larger sample size are required to assess the variables and their interconnectedness.

Fifth, based on the integrated information from literature, it seems variations in BSE training approaches can be very beneficial in achieving effective results. For example, blended learning could be an example of combining both face-to-face and online approaches to provide more flexibility and engagement. In addition, having a follow-up system in place can be paramount to a more sustainable result in BSE training.

### Strengths and limitations

This study has some key strengths. First, this study is an interventional study comparing two educational training, adding knowledge to the current descriptive research and those with no comparison. Second, the main criteria for the population was to approach those who have never participated in any BSE to ensure the effectiveness of the two training interventions. Third, the participants were selected randomly, and the interventions were blinded (those who did the delivery and assessments were blinded to participants and researchers). Fourth, the content development across the two interventions was quite similar, allowing us to focus better on the training techniques, including online and face-to-face and their differences.

This study has some fundamental limitations. First, although focusing on participants without any previous training is one of our work's strengths, it can also be a limitation as it limits us to some specific population (e.g., low socioeconomic status populations). Second, although the sampling was random because the targeted population was those who have never participated in any BSE, we came across a high range of people with a lower level of education and employment. Third, although we measured the participants' performance on BSE with a checklist in the pre-test and post-test, it was impossible to measure their actual behaviour precisely. Although this study was conducted in one of the biggest cities in Iran (Shiraz), the generalization of the findings needs to happen cautiously. This study was conducted in one of the biggest cities in Iran (Shiraz); the findings' generalization needs to happen cautiously.

## Data Availability

The datasets used and/or analyzed during the current study are available from the corresponding author on reasonable request.

## Abbreviations

SUMS: Shiraz University of Medical Sciences
BSE: Breast self-examination
BC: Breast cacer
